# Demand side management using optimization strategies for efficient electric vehicle load management in modern power grids

**DOI:** 10.1371/journal.pone.0300803

**Published:** 2024-03-21

**Authors:** Manoj Kumar V., Bharatiraja Chokkalingam, Devakirubakaran S.

**Affiliations:** 1 Department of Electrical and Electronics Engineering, SRM Institute of Science and Technology, Kattankulathur, India; 2 Department of Mechanical Engineering, SRM Institute of Science and Technology, Kattankulathur, India; University of Lagos Faculty of Engineering, NIGERIA

## Abstract

The Electric Vehicle (EV) landscape has witnessed unprecedented growth in recent years. The integration of EVs into the grid has increased the demand for power while maintaining the grid’s balance and efficiency. Demand Side Management (DSM) plays a pivotal role in this system, ensuring that the grid can accommodate the additional load demand without compromising stability or necessitating costly infrastructure upgrades. In this work, a DSM algorithm has been developed with appropriate objective functions and necessary constraints, including the EV load, distributed generation from Solar Photo Voltaic (PV), and Battery Energy Storage Systems. The objective functions are constructed using various optimization strategies, such as the Bat Optimization Algorithm (BOA), African Vulture Optimization (AVOA), Cuckoo Search Algorithm, Chaotic Harris Hawk Optimization (CHHO), Chaotic-based Interactive Autodidact School (CIAS) algorithm, and Slime Mould Algorithm (SMA). This algorithm-based DSM method is simulated using MATLAB/Simulink in different cases and loads, such as residential and Information Technology (IT) sector loads. The results show that the peak load has been reduced from 4.5 MW to 2.6 MW, and the minimum load has been raised from 0.5 MW to 1.2 MW, successfully reducing the gap between peak and low points. Additionally, the performance of each algorithm was compared in terms of the difference between peak and valley points, computation time, and convergence rate to achieve the best fitness value.

## 1. Introduction

The Electric Vehicle (EV) landscape has witnessed unprecedented growth over recent years. Propelled by technological advancements, environmental concerns, and supportive policies, EVs are swiftly becoming a mainstay on roads globally. This transition represents not just a shift in consumer preference but also heralds a significant transformation in energy consumption patterns and the demands placed on the electrical grid [1]. The average EV requires a significant amount of energy to charge. A typical EV might need approximately 30 kWh of electricity for every 100 miles of range. To put this into perspective, the average American household consumes about 877 kWh per month. If even a fraction of Existing Internal Combustion based car owner’s switches to EVs, the additional load could be substantial. For instance, if 10% of the driving population adopted electric vehicles and each of those EVs consumed an additional 300 kWh per month for charging, this could translate to millions of extra kilowatt-hours needed monthly [2]. This demand places an additional burden on our power grids, which must be prepared to supply this energy reliably and sustainably [3]. The impact of EVs on daily load demand is both profound and multifaceted. The most noticeable effect is on peak demand times, typically early evening when people return home and plug in their vehicles for charging. Without intervention, this could exacerbate the evening peak load, potentially requiring utilities to activate additional, often less efficient, power plants to meet the demand. thus, raising concerns about grid stability, electricity prices, and environmental impacts [4]. The load profile of a grid–that is, the typical pattern of electricity usage over time is being reshaped by EVs. Traditionally, residential load profiles showed peaks in the morning and evening, corresponding to when people were most likely to be at home and using electricity. However, as EVs become more common, these profiles become steeper with sharper peaks, unless smart charging strategies or demand response programs are implemented. The ’load curve’ which graphically represents the variation of electrical load throughout the day, thus takes on a new shape in the age of EVs. It becomes not just a tool for observing current energy use but a map for predicting and managing the future, especially as the proportion of EVs continues to rise [5]. In some cases, the EV supports the grid during the peak load operating conditions by using vehicle to grid (V2G) technology. This setup can be constructed using distributed Flexible AC Transmission System(FACTS)based switched filter compensator (SFC) and a dynamic multi-level error-driven control strategy for efficient utilization of vehicle-to-grid battery chargers [6].

**Fig 1:**
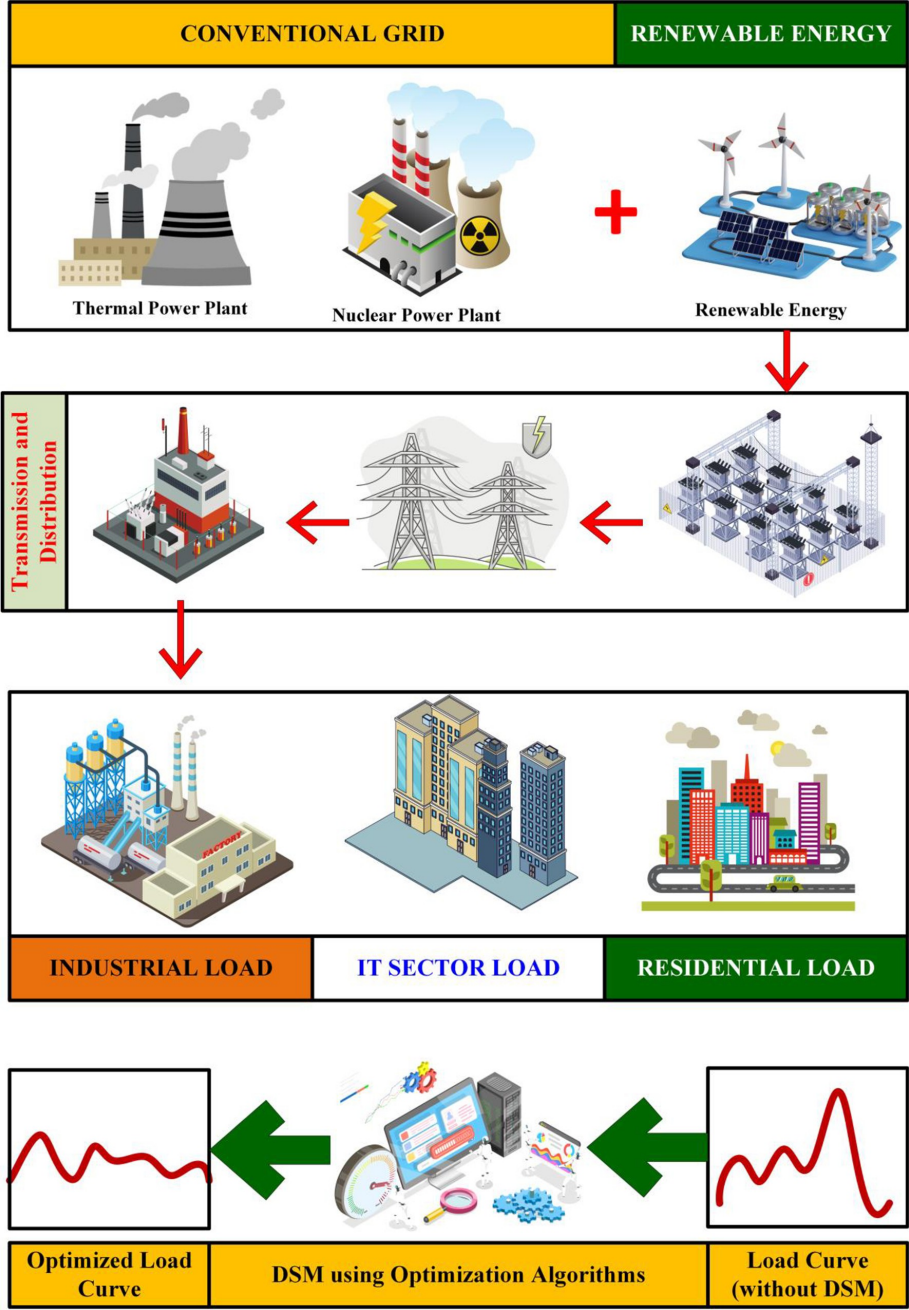
Demand side management system.

Demand Side Management (DSM) plays a pivotal role in this system by managing the demand for power in a way that maintains the grid’s balance and efficiency [7]. The integration of EVs increases the load demand, especially during peak hours, which can lead to overloads and even blackouts. It’s vital to understand how this affects the grid’s load profile and to find ways to mitigate any negative impacts. Along with these inclusion of the renewable energy sources, particularly solar PV systems are reducing the stress of grid caused by the EVs [8, 9]. As shown in the Fig 1, Implementing DSM strategies helps in smoothing the load curve, ensuring that the grid can accommodate the additional demand from EVs without compromising on stability or necessitating costly infrastructure upgrades. Strategies like time-of-use tariffs and smart charging are key to managing the load. Time-of-use tariffs encourage charging during off-peak hours, while smart charging systems can automatically adjust charging times based on grid demand. Case studies from around the world demonstrate the efficacy of DSM in integrating EVs into the grid. They provide real-world evidence of how such strategies can be successfully implemented. The role of consumers is critical in DSM. Incentives and educational programs are essential to encourage smart energy usage that aligns with DSM objectives. DSM implementation is not without its challenges. Technical, logistical, and consumer comfort considerations must be addressed to create a successful strategy. Many of the tools were already available such as extra-tree, bagging k-nearest neighbors (KNN), voting regressor, random forest, and boosting algorithms for monitoring the faults and improving the performance of the systems [10–12]. Along with that, the DSM must access the available charge scheduling algorithms employed for the charge allocation system of EVs. These algorithms will be helpful for DSM to schedule the EVs effectively [3, 13].

Innovative algorithms have been the cornerstone of effective DSM strategies, allowing to optimize energy usage without compromising the comfort of the end-users [14–16]. The Bender algorithm has been at the forefront of these strategies, enabling sophisticated planning and efficient resource allocation within the energy sector. Its design caters to the dynamic nature of energy demands, ensuring that energy distribution is both practical and cost-effective [17]. Genetic Algorithms (GA) have been employed to schedule household appliances in a way that minimizes energy costs [18]. These algorithms mimic the process of natural selection to find the most efficient schedule for appliance usage. However, the complexity of these GAs often leads to high computational loads, which can be a hurdle, especially when user convenience is a secondary consideration. The challenge lies in creating a system that not only cuts down on energy costs but also prioritizes user comfort. Achieving this balance is the holy grail of energy management [19]. A novel approach is the grey wolf accretive satisfaction algorithm, a strategy that puts user comfort at its core while striving for the lowest possible energy expenditure. This algorithm takes inspiration from the hunting behavior of grey wolves, aiming to provide a smart and adaptive energy management solution that aligns with the user’s lifestyle and preferences [20, 21].

Alongside, the Candidate Solution Updating Algorithm (CSUA) has been introduced. Its goal is crystal clear: minimize the time users spend waiting on appliances without diminishing their level of comfort. CSUA is a testament to the user-centric design in DSM, ensuring that energy savings and user convenience go hand-in-hand [22]. The collaboration between modified differential evolution and grey wolf optimization leads to a sophisticated model of energy management, aiming to slash peak energy usage and overall electricity costs [23]. This fusion is a powerhouse, driving forward the efficiency of energy systems and setting new benchmarks for what is possible in DSM. Advanced strategies such as load clipping and shifting have been developed, with simulations carried out in MATLAB/Simulink and further optimized using an Artificial Neural Network (ANN) algorithm [24]. The ANN algorithm refines these strategies, ensuring that they are not only effective but also adaptable to a myriad of energy scenarios and user behaviors.

The inclusion of the EV loads into the grid, makes an impact on the load curve. This affects the performance of the normal and optimization-based DSMsystems. This additional load reduces the accuracy and effectiveness of the objective functions and the constraints. In this work, the proper objective functions with necessary constraints with the inclusion of the EV load has been defined. Along with this, the objective function has been constructed with different optimization algorithms such as Bat Optimization Algorithm (BOA), African Vulture Optimization Algorithm (AVOA), Adaptive Neuro-Fuzzy Inference System (ANFIS), Cuckoo Search (CS) Algorithm, Chaotic Harris Hawk Optimization (CHHO), Chaotic-based Interactive Autodidact School (CIAS) algorithm and, Slime Mould Algorithm (SMA). The constraints and the execution of all these algorithms for the defined demographic area has been executed in all possible considerations.

The contribution of the proposed work are as follows,

- The research focuses on DSM as a key solution for managing the extra load on electrical grids due to EV charging, especially during peak hours.
- This work introduces various optimization algorithms for efficient energy usage optimization in DSM, considering the additional load from EVs.
- It examines how EV charging affects the grid’s load curve and adapts DSM strategies to maintain grid efficiency and stability.
- The inclusion of solar power generation supports the energy demand on peak hours and reduces the stress on the grid.
- It highlights the potential of DSM strategies in grid management with EV integration and suggests incorporating renewable energy sources.

The organization of the works is as follows: Section 2 discusses about the defined demographic area and the corresponding load profiles, Section 3 discusses about the objective function, constrains and the development of six different optimization methods, Section 4 discusses about the evaluation of the proposed DSM methods in all possible load variations and its effectiveness on this circumstance with necessary results, Section 5 presents the discussion over the performance of each methods and section 6 concludes the manuscript.

## 2. Problem statement

The shift to EVs poses challenges for optimizing grid consumption and integrating renewable energy. EV adoption increases residential energy loads and peak demands, requiring grid expansion. The challenge is adapting the grid for EVs, ensuring a smooth transition and handling increased demands. DSM plays a pivotal role, offering effective solutions to balance and optimize energy usage for a sustainable future. It promotes decentralized and efficient operation of appliances and EV charging. It addresses grid issues, plays a crucial role in EV integration, and ensures grid stability without costly infrastructure upgrades. DSM stabilizes the grid during variable EV demands by optimizing charging timing, reducing peak hour burdens. It also brings complexity due to the high variability of EV charging loads, the spatial and temporal variations in energy demand, and the challenges of integrating intermittent renewable energy sources whereas, traditional optimization methods often fall short in managing these complexities, which include peak load management, unpredictable user behavior, network constraints, and the need for advanced communication and control technologies. To realize this optimal DSM, a range of innovative heuristic algorithms are explored for global optimization challenges inherent in DSM. These include the BOA, known for its efficiency and simplicity, AVOA is noted for its robustness, CS algorithm is recognized for fast convergence, CHHO is appreciated for its adaptive search capability, CIAS algorithm is valued for its interactive learning approach and SMA is distinguished for its ability to avoid local optima. These heuristic algorithms are specifically chosen for their unique strengths to effectively manage the integration of EVs and other dynamic loads into the grid, ensuring a sustainable and efficient energy system. Ultimately, this work aims to harness the full capabilities of DSM, employing these advanced algorithms to effectively manage the integration of EVs and other dynamic loads into the grid. The simulation is designed to ensure fairness among all users, taking into account both the overall cost reduction and individual cost reduction. The goal is to pave the way for a more sustainable, reliable, and efficient energy system, bridging the gap between increasing energy demands and the imperative for environmental stewardship.

## 3. Structure of the proposed system

**Fig 2:**
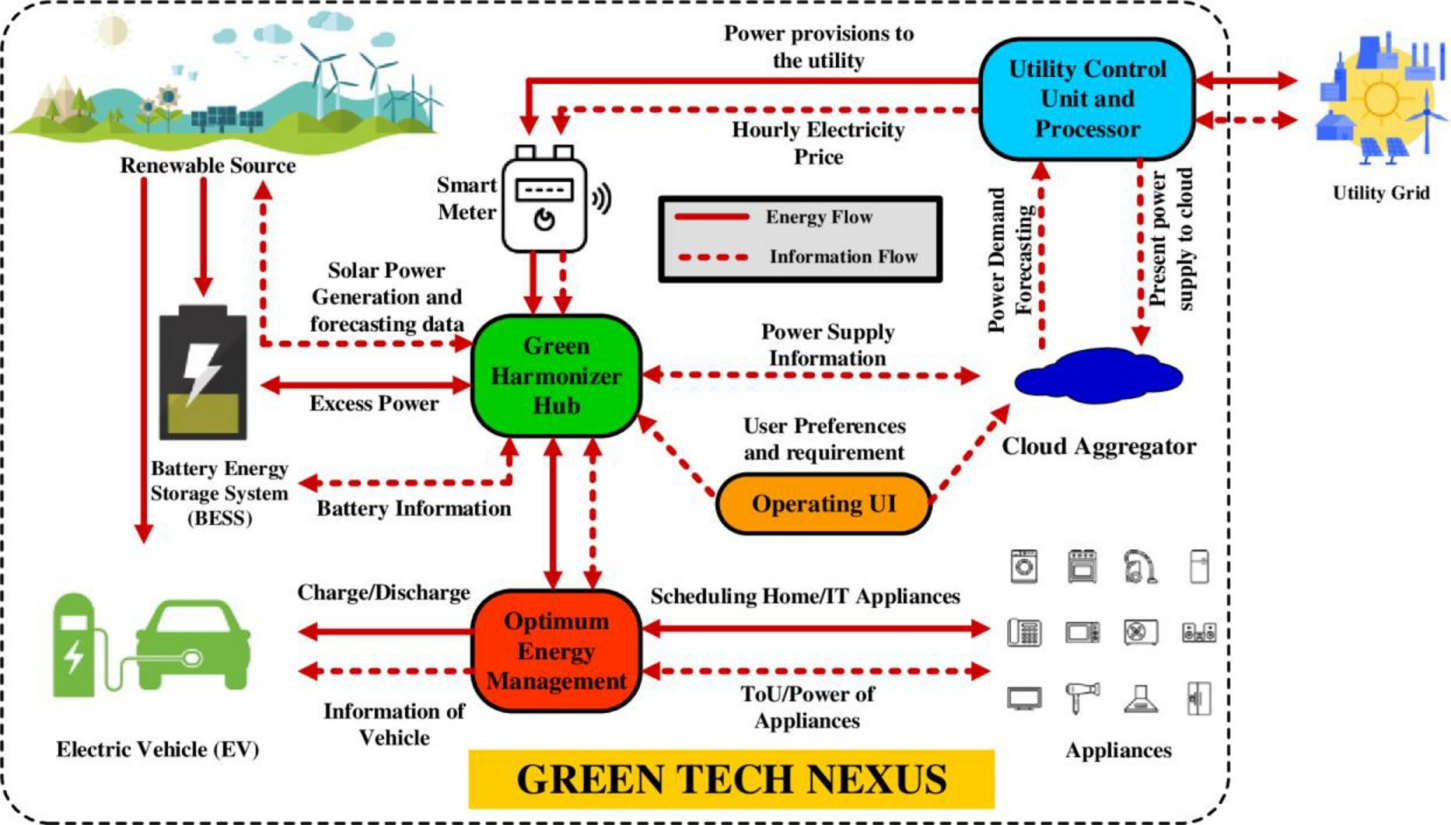
Graphical representation of the proposed input output model of GN system.

The architectural framework of GreenTech Nexus (GN), an integrated model of mixed-use area for sustainable living and business-oriented technological advancement, the graphical representation of the proposed model is showcased in Fig 2. GN connects with an aggregator to transmit power demands to the grid, including energy consumption by residential and IT sectors for the next day. The detailed description about the proposed system is briefed below.

### 3.1 Defined input and output model

The input model of the GN system is ingeniously designed to efficiently handle energy demands, particularly in the residential and IT sectors. At its core, the system is geared towards shifting energy consumption to off-peak hours. GN is outfitted [4] with an array of components including smart meters for real-time energy tracking, user interfaces for interactive management, scheduler modules for energy allocation, Home Energy Management (HEM) systems for optimizing efficiency, smart appliances that adapt to varying energy supplies, photovoltaic (PV) panels for sustainable energy generation, Battery Energy Storage Systems (BESS) for storing electricity, and EVs as both a load and a potential energy reservoir. This is facilitated by the integration of smart meters and a comprehensive communication network, which plays a pivotal role in aggregating and transmitting power demands from local areas to the grid. The inclusion of the aggregator further enhances this process, enabling the GN system to predict and manage the consecutive day’s energy consumption in a more efficient manner.

The output model of the GN system is characterized by its ability to maintain a consistent and efficient power supply. This is achieved through the main power contactor, which acts as an automatic switch essential for the smooth transmission of electricity across the network. The system’s efficient distribution of power, guided by the GreenHarmonizer Hub (GH), ensures that energy is utilized in the most effective manner, leading to a sustainable and balanced energy ecosystem. This integration of advanced scheduling and management capabilities makes the GN system an adept at catering the dynamic needs of both living and business environments.

GH is the central to the input and output model of GN system’s energy management. This innovative component acts as a scheduler, taking charge of decision-making and scheduling the energy usage in both smart homes and IT facilities. The GN infrastructure also includes a distribution board, crucial for dividing the power supply into various circuits and effectively distributes flexi (shiftable) energy loads from the constant (non-shiftable) one. The GH emits energy price alerts and identifies major energy-consuming appliances, optimizing energy usage by orchestrating the timings of household appliances. It receives real-time pricing indicators from the central grid via a smart meter, enabling fine-tuning of energy consumption each hour. The Smart Scheduler adjusts peak electricity consumption, shifting it to lower-cost periods, reducing expenditures, and balances the demand on the power grid. The GH maximizes energy efficiency by aligning consumption with optimal times, intelligently distributing energy, and optimizing overall network usage.

### 3.2 Cloud-aggregated energy optimization

The GreenTech Nexus (GN) system, with its cloud-enabled algorithm within the GreenHarmonizer Hub, plays a crucial role in optimizing energy usage across both smart homes and IT sectors. Its integration of cloud technology and advanced algorithms for optimization demonstrates its capacity to handle complex and dynamic energy management tasks. The core function of the GreenHarmonizer Hub is to process data on energy consumption and price fluctuations in real-time. This data is then processed to modify the energy consumption strategy. By shifting energy consumption from high-cost peak hours to more economical off-peak periods, the system not only reduces energy cost but also contributes to balanced energy distribution in the grid. The adaptability of the GreenHarmonizer Hub to varying energy needs and market conditions highlights its efficiency and makes it a crucial component in energy management systems.

The GN system’s integration with a cloud-based DSM system involves using smart meters and sensors for data collection. This data is crucial for understanding and optimizing energy use. The optimization engine, powered by meta heuristic optimization algorithms, works in the cloud to tailor energy consumption strategies. This engine takes into account specific objectives and constraints, ensuring optimal energy management. This feature enhances user engagement and control. by providing interactive dashboards that allow users in both residential and IT sectors to actively manage their energy consumption. The GN system’s ability to integrate with existing home automation systems and IT infrastructures makes it versatile and user-friendly. Upholding rigorous security standards and compliance measures ensures data integrity and adherence to regulatory requirements. This aspect is critical in maintaining user trust and system reliability. Regular monitoring and maintenance of the GN system ensures that it operates at optimal efficiency, adapting to changing conditions and requirements.

### 3.3 Proposed methodology for analyzing energy consumption in DSM framework

**Fig 3:**
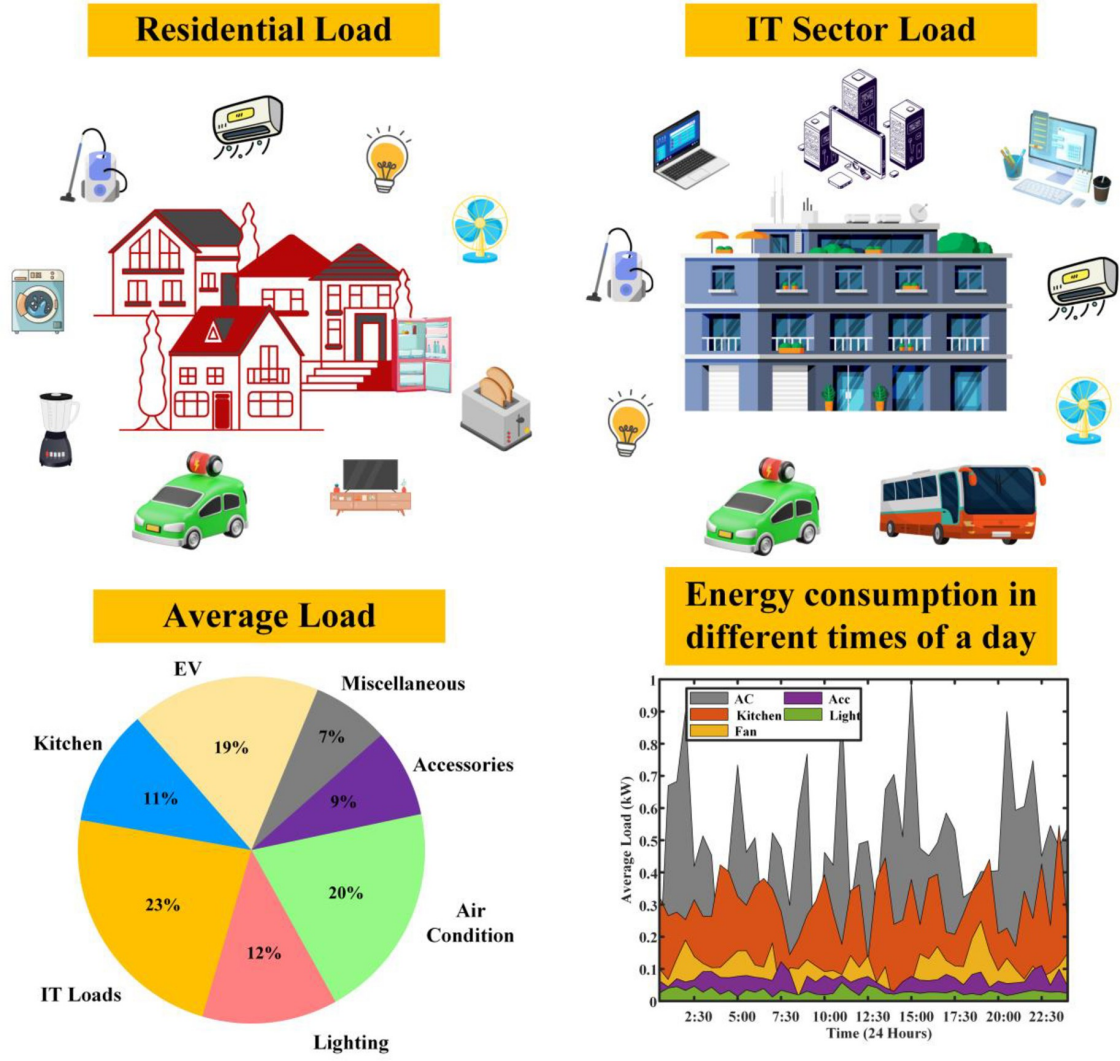
Different loads and load consumption of the demographic area.

The proposed methodology for understanding the energy consumption patterns in the GN system simulation involves a comprehensive analysis of both the residential and commercial sectors, specifically focusing on a residential community of 6,000 houses and an IT sector company with 3,000 employees. This approach takes into account the unique consumption characteristics of these two sectors and explores the implications of integrating coordinated EVs charging with operational performance of grid [7] into their respective ecosystems. The complexity of their electricity usage patterns forms a multi-dimensional puzzle, one that energy analysts and policymakers strive to solve. This discourse presents a detailed analysis of the load profiles of a residential community, delving into the residential landscape, different consideration of a variety of living spaces: 1 Bedroom-Hall-Kitchen (BHK), 2 BHK, and 3 BHK houses, each with distinct occupancy rates. The demographic diversity confirms in the electric load patterns, showing a representation of electricity use that’s as varied as the lives of the inhabitants. Within these dwellings, a spectrum of appliances from essential to additional buzz with activity. As in the Fig 3, probability of each appliance’s presence is not uniform; some homes brim with gadgets, while others subscribe to minimalism, affecting the overall load profile. The possibility the residential loads considered in this works are, lights (15W Per Person (PP) always present), fan (70W PP, always present), refrigerator 150W (always present), Air Conditioner (AC) (1.5kW per room, 70% probability), Television (TV) (120W per room, 90% probability), Microwave (1kW, 30% probability), mixer (500W, 80% probability), Geyser (2kW, 60% probability), Room Heater: 1kW (only in winter), 40% probability, Washing Machine (500W, 70% probability).

Appliance utilization doesn’t adhere to a fixed schedule. Usage times are randomized, with a touch of logic applied to certain devices like washing machines, their cycles intertwined with occupancy levels, contributing to a dynamic load profile that’s as unpredictable as it is real. Amidst the energy consumption, solar panels emerge as beacons of sustainability on 20% of the rooftops, offsetting grid electricity demand during the sunlit hours, casting a green glow on the load profile. The daily exodus of working occupants leaves a distinct imprint on the load profile, with adjusted electricity usage reflecting their absence during office hours, a silent indication to the fade and flow of working life. Transitioning to the commercial sector, the IT sector’s energy blueprint is drawn with precise lines: a computer for each of the 3,000 employees, servers humming at the heart of operations, and an array of other appliances contributing to the load.

**Fig 4:**
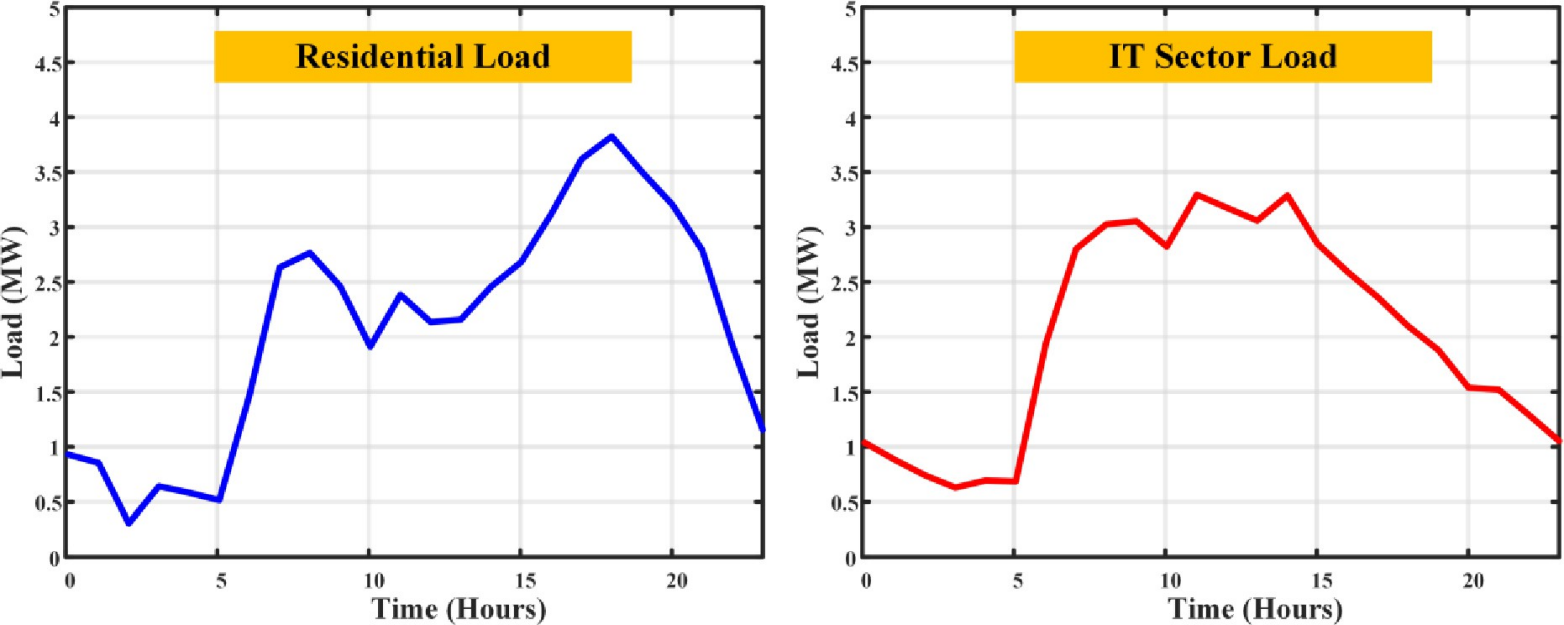
Load curves of residential and IT sector loads without DSM.

Every piece of equipment draws power: computers (150W each), servers (an average of 500W), lighting (20W per employee), and air conditioning calibrated to space size (1kW per 100 sq.m.), with a symphony of additional appliances adding their chords to the energy melody. The load profile fluctuates with the day’s rhythm a crescendo during working hours as computers, lights, and ACs perform in unison, and a decrescendo into the night with reduced lighting and AC use, though servers maintain their vigil. The output is an estimation of total load, a pulse that quickens and slows with the operational heartbeat of an IT company, providing a granular view of commercial electricity use. Merging the residential and IT sector load profiles unveils a comprehensive panorama of electricity demands, highlighting the contrasting usage patterns in the dwellings of daily life and the hubs of technological innovation. The total load profile of the given demographic area has been simulated as shown in Fig 4.

**Fig 5:**
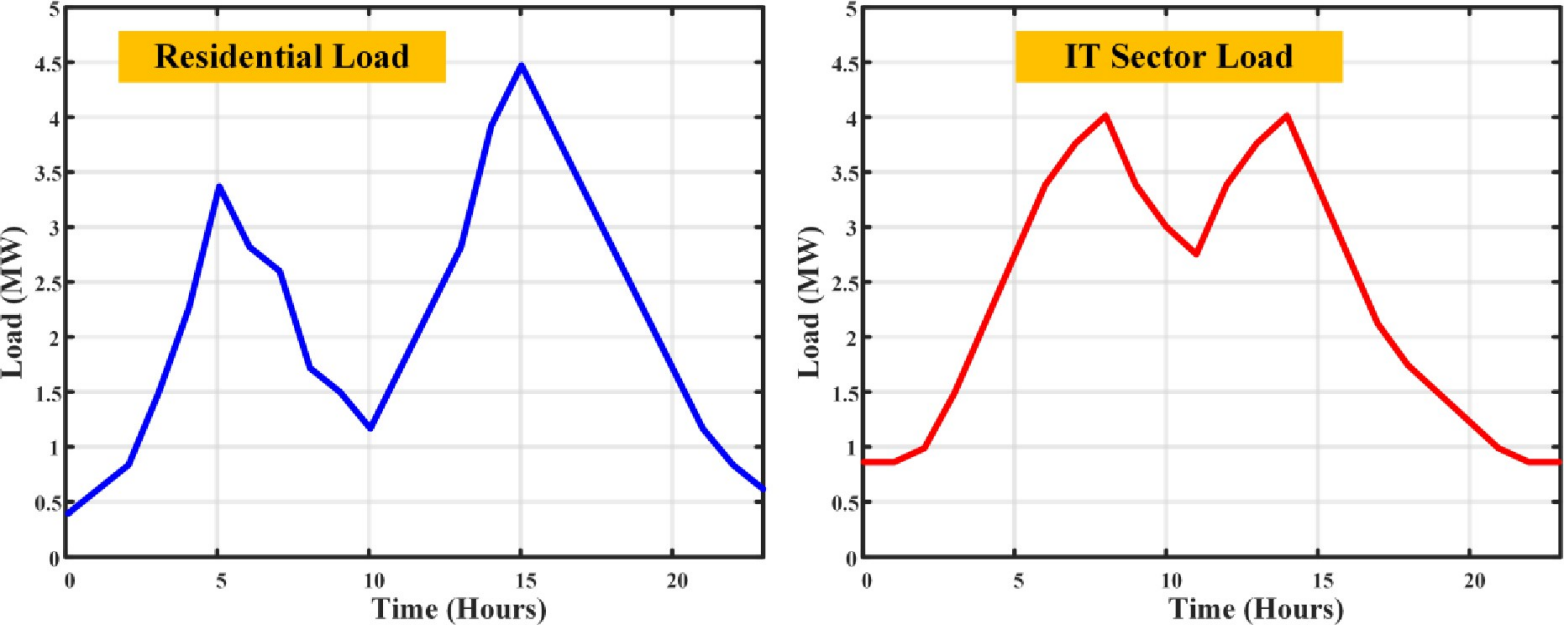
Load curves of residential and IT sector loads with the inclusion of EV loads.

The chances of EV presence vary with house size, from the modest 1 BHK to the more spacious 3 BHK, each with their probabilities of housing zero, one, or even two EVs, adding another layer of complexity to the electricity consumption tapestry. This EV load creates an impact on the normal load profile. There are two several concerns need to be addressed such as the load demand of the area and the dependency of grid will be increased, also uneven charge scheduling of the EV causes increases the peak demand of the system. The load distribution on the load curve with the inclusion of EV load has been plotted as shown in Fig 5.

## 4. Problem formulation

The developed equations for the GN system simulation deliver an in-depth and quantifiable method for analyzing the intricate energy usage dynamics present in both residential and commercial settings, notably in a typical residential neighborhood and an IT company. These formulations are intricately designed to encapsulate distinct patterns of energy consumption arising from several key factors such as the utilization of various appliances, the frequency of their use, occupancy rates, the number of operational hours, as well as the integration aspects of EVs and solar energy systems.

### 4.1 Mathematical model for energy consumption

The comprehensive mathematical model manages energy consumption by distinguishing between flexible and non-flexible appliances, enabling dynamic energy management. It calculates each appliance’s energy consumption based on power rating, operational duration, and likelihood of usage, determining the total energy consumption of the household. By categorizing appliances into flexible and non-flexible, the model offers a nuanced understanding of household energy dynamics, allowing for the optimization of energy usage to align with cost-saving opportunities and demand-response initiatives.

For flexible appliances, the model incorporates a layer of flexibility, accounting for the likelihood that an appliance may be used during off-peak hours when energy rates are lower. This is represented mathematically by the inclusion of a binary decision variable in the Eq 1 for flexible appliances $E_{t,R_{shift}}$, hinges on a set of parameters, the power rating P_r​,_ which quantifies the energy an appliance uses when active; the operational hours H_t,r_​, reflecting the duration of use within the chosen time slot t and a binary decision variable b_t.r_​, indicative of whether the appliance is turned on or off. This decision variable is particularly important as it allows for the scheduling flexibility of these appliances, enabling them to be turned on during off-peak energy hours for cost-saving purposes or when renewable energy availability is high. The usage probability variable, U_t,r_ ​further refines this by accounting for user behavior such as the tendency to run dishwashers overnight or automated scheduling systems that optimize for energy savings.


$$E_{t,R_{\mathrm{shift}}}=\sum_{r\in R_{\mathrm{shift}}}P_{r}\times H_{t,r}\times b_{t,r}\times U_{t,r}$$ (1)

Conversely in Eq 2, non-flexible appliances are those with fixed usage patterns, such as refrigerators and security systems, which are essential for the household’s functionality and typically operate continuously. These appliances are captured in the model without the need for a binary decision variable, as their operation is constant. Instead, their energy consumption is a straightforward product of their power rating and operational hours, $E_{t,R_{nonshift}}$, reflecting their steady and predictable demand on the energy system.


$$E_{t,R_{\mathrm{nonshift}}}=\sum_{r\in R_{\mathrm{nonshift}}}P_{r}\times H_{t,r}\times C_{r}$$ (2)

The model’s separation of appliances into these two categories allows for a targeted approach to managing energy consumption [15]. For instance, during peak demand hours, energy usage can be shifted away from flexible appliances, reducing strain on the grid and household energy costs. Meanwhile, the consistent load of non-flexible appliances provides a baseline for energy needs, ensuring that essential services remain uninterrupted.


$$R_{\mathrm{base}(t)}=E_{t,R_{\mathrm{shift}}}+E_{t,R_{\mathrm{nonshift}}}$$ (3)

The residential base load *R*_*base*(*t*)_, is calculated by summing the energy usage of flexible and non-flexible appliances. The Eq 3 represents the total energy requirement for the household at any given time, considering both flexible and steady appliance demands.


$$R_{\mathrm{base}(t)}=\sum_{r\in R_{\mathrm{shift}}}P_{r}\times H_{t,r}\times b_{t,r}\times U_{t,r}+\sum_{r\in R_{\mathrm{nonshift}}}P_{r}\times H_{t,r}\times C_{r}$$ (4)

The energy model for an IT sector company takes into account the constant demand from critical infrastructure, such as servers and networking equipment, which are non-flexible due to their 24/7 operational requirements. These elements are essential for the uninterrupted functioning of the company and typically do not have the flexibility to be turned off or operated at reduced capacity. The power consumption for these non-flexible components can be denoted by P_c_ ​,​ and is multiplied by their operational hours ​ H_t.c_ ​, which, for non-flexible loads, are consistent across all time slots. On the other hand, flexible loads in an IT company might include workstations, certain office equipment, and possibly discretionary use of climate control systems, which can be adjusted based on occupancy and need. These flexible loads allow for energy management strategies, such as scheduling intensive computational tasks during off-peak hours or adjusting air conditioning when areas of the office are unoccupied. The equation for the base load of IT sector company’s *I*_*base*(*t*)_ would be represented as follows in Eq 5.


$$I_{\mathrm{base}(t)}=\sum_{c\in C_{\mathrm{shift}}}P_{c}\times H_{t,c}\times b_{t,c}\times U_{t,c}+\sum_{r\in C_{\mathrm{nonshift}}}P_{c}\times H_{t,c}$$ (5)

To formulate the problem realistically, the Eq 6 represents S_solar_(t) evaluates the impact of renewable energy and solar implementation, considering technology and the environment. It incorporates the installed capacity of solar panels (C_solar_) and their efficiency rate (η_solar_) to account for real-world factors affecting energy output. Additionally, the equation includes the variable S_t_, representing sunlight availability influenced by time, weather, and seasons, and Δt, representing the duration for solar energy conversion.


$$S_{\mathrm{solar}}(t)=C_{\mathrm{solar}}\times \eta_{\mathrm{solar}}\times S_{t}\times \Delta t$$ (6)

The Eq 7 represents for EV charging load plays a pivotal role in understanding the growing influence of EVs on energy consumption patterns in both residential and commercial sectors. *E*_*EV*_(*t*) equation plays a crucial role in quantifying the total EV charging load at a specific time t, and it is an integral component in understanding the evolving landscape of energy consumption in both residential and commercial sectors.


$$E_{EV}(t)=\sum_{EV=1}^{N_{EV}}P_{EV}\times c_{t,EV}$$ (7)

This Eq 7, expressed is designed to calculate the combined power demand of EVs that are actively charging at a given moment. In this formulation, *R*_*EV*_(*t*) stands for the total EV charging load at time t. The equation takes into account each EV within the scope of the analysis, ranging from the first EV to the N_EV_^th^ EV, where N_EV ​_ represents the total number of EVs that could potentially be charging at that time. This count is vital as it reflects the scale of EV charging infrastructure and usage within the area under consideration, whether it’s a residential neighborhood or a commercial parking facility.

The power rating of each EV charger (P_EV_) is a crucial factor in the equation, varying based on vehicle and charger type. A binary variable c_t,EV_ indicates the charging status of each EV at time t, allowing for dynamic representation of charging activities. The variable c_t,EV_ is set to 1 if an EV is being charged during the time slot, and 0 if it is not, representing the dynamic nature of EV charging activities over time.

### 4.2 Residential and IT sector load

The total loads for residential and IT sectors is expressed in Eqs 8 and 9 which includes the base loads *R*(*t*) and *I*(*t*) with EV load R_EV_(t) and I_EV_(t), and the solar panel generation S_solar_(t) and ev intrusion E_EV_(*t*) can be expressed as,

$$R(t)=c+E_{EV}(t)-S_{solar}(t)$$ (8)

$$I(t)=I_{\mathrm{base}}(t)+E_{EV}(t)-S_{\mathrm{solar}}(t)$$ (9)

The inclusion of EV load in the daily load profile of the grid operation increases the complexity. The DSM is one of the solutions to reduce the problems that arise due to the EV loads. The existing DSM methods constructed with the objective functions and constraints without the EV loads, where the inclusion of create an impact on the objective function as well as on the designed constraints. The revised objective function F can be expressed as in Eq 10,

$$\mathrm{Minimize}\;D_{\mathrm{peak}}=\max_{t\in T}\{R(t)+I(t)\}$$ (10)

where D_peak_ is the peak demand during the time period T, and R(t) and I(t) represent the total residential and IT sector loads at time (t), respectively.

### 4.3 Constraints

Constraints in demand side energy management and grid optimization by setting defined rules and conditions for the system operation. These constraints help to balance different requirements, optimize the use of renewable energy sources, meet the operational needs of EVs, ensure efficient electricity usage, maintain grid stability, and prevent overloading. By adhering to these constraints, energy system efficiency and sustainability can be improved, allowing for the maximization of available resources while meeting consumer needs and maintaining grid integrity.

#### EV charging schedule constraints

EVs are not to be charged during peak demand hours, which need to be defined as Eq 11,

$$R_{EV}(t)=I_{EV}(t)=0\;\mathrm{for}t\in \mathrm{Peak}\;\mathrm{Hours}$$ (11)

#### Minimum state of charge for EVs

EVs must still meet a minimum state of charge by a certain time is defined as Eq 12,

$$SoC_{EV}^{min}\leq SoC_{EV}(t)$$ (12)

#### Non-peak charging windows

EVs should be charged during non-peak hours and Emergency Hours which explicitly defined in the Eqs 13 and 14,

$$R_{EV}(t),I_{EV}(t)>0\;\mathrm{for}t\notin \mathrm{Peak}\;\mathrm{Hours}$$ (13)

$$R_{EV}(t),I_{EV}(t)=R_{EV_{Emg}}(t),I_{EV_{Emg}}(t)\;\mathrm{for}t\in \mathrm{Emergencyhours}$$ (14)

#### Solar generation constraints

The contribution of solar generation to the load must be accounted for fitness function is defined in the Eq 15.


$$0\leq R_{\mathrm{solar}}(t)\leq R_{\mathrm{solar}}^{\max}(t)$$ (15)

#### Load shifting

Loads other than EV charging can be shifted if necessary by following below Eq 16.


$$R_{\mathrm{base}}(t),I_{\mathrm{base}}(t)\leq L_{\max}^{\mathrm{shif}t}$$ (16)

#### Maximum and minimum load levels

Eq 17 is defined to the Loads, which must stay within certain levels to ensure functionality and comfort of the grid operation.


$$L_{\min}\leq R(t),I(t)\leq L_{\max}$$ (17)

#### Total daily energy consumption

The DSM should not result in a total energy consumption that exceeds predefined targets is explicitly constrain to the Eqs 18 and 19.


$$\overset{T}{\underset{t=1}{\sum }}R(t)\leq E_{R,\mathrm{target}}$$ (18)

$$\overset{T}{\underset{t=1}{\sum }}I(t)\leq E_{I,\mathrm{target}}$$ (19)

### 4.4 Mathematical expression for the EV charging rescheduling

Let’s define P as the set of peak hours during the day to the defined Eq 20 for EV charging rescheduling, E is the emergency where vehicle needs to be charged and NP as the set of non-peak hours. The constraint for EV charging rescheduling can be expressed as:

$$R_{EV}(t)=I_{EV}(t)=\{\begin{array}{ll} 0 & \mathrm{if}t\in P\\ R_{EV}^{\mathrm{sched}}(t) & \mathrm{if}t\in E\\ R_{EV}^{sched}(t) & \mathrm{if}t\in NP \end{array}$$ (20)

**Fig 6:**
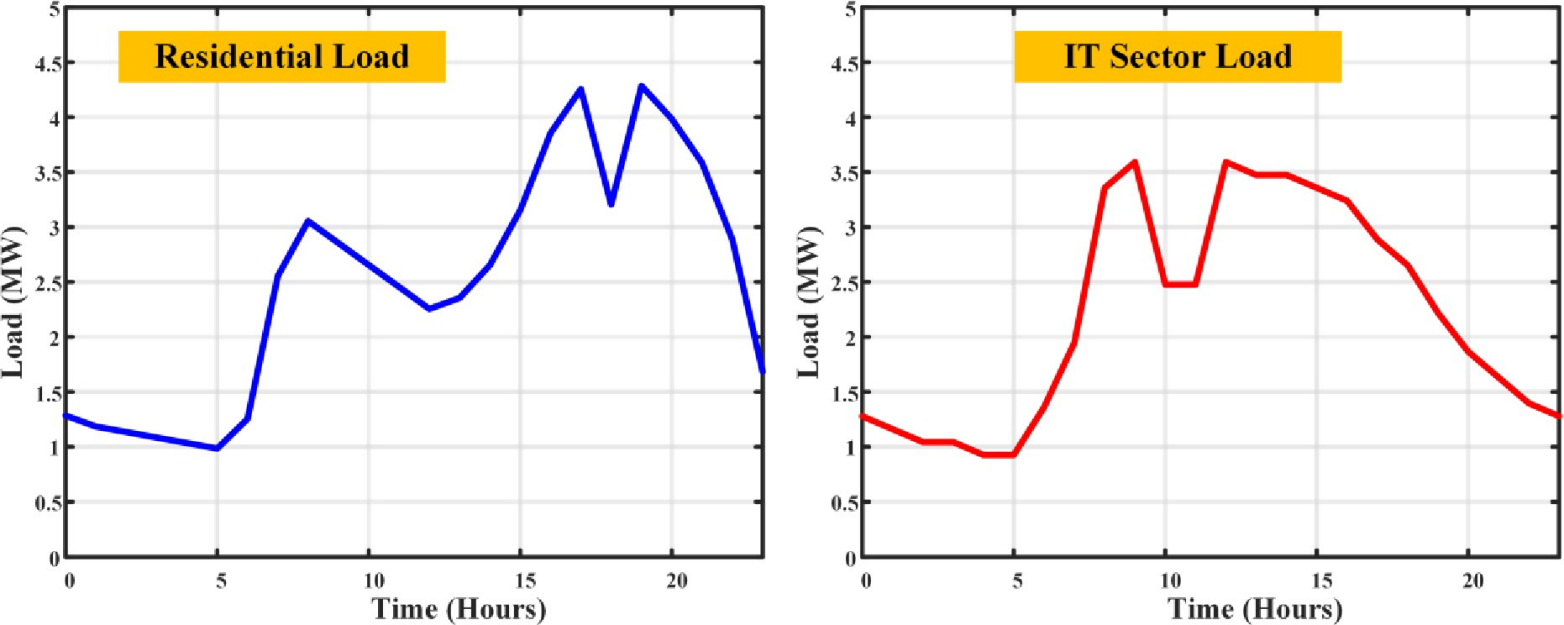
Load curves of residential and IT sector loads with DSM.

Where $R_{EV}^{sched}(t)$ and $I_{EV}^{sched}(t)$ are the scheduled EV charging rates during non-peak hours, which are determined by the available charging time, the energy requirement of the EVs, and the EV charging infrastructure’s capacity. By the execution of this objective function and the constraint the peak demand has been compensated by valley filling. The load curve obtained after the implementation of the DSM algorithm is shown in Fig 6.

**Fig 7:**
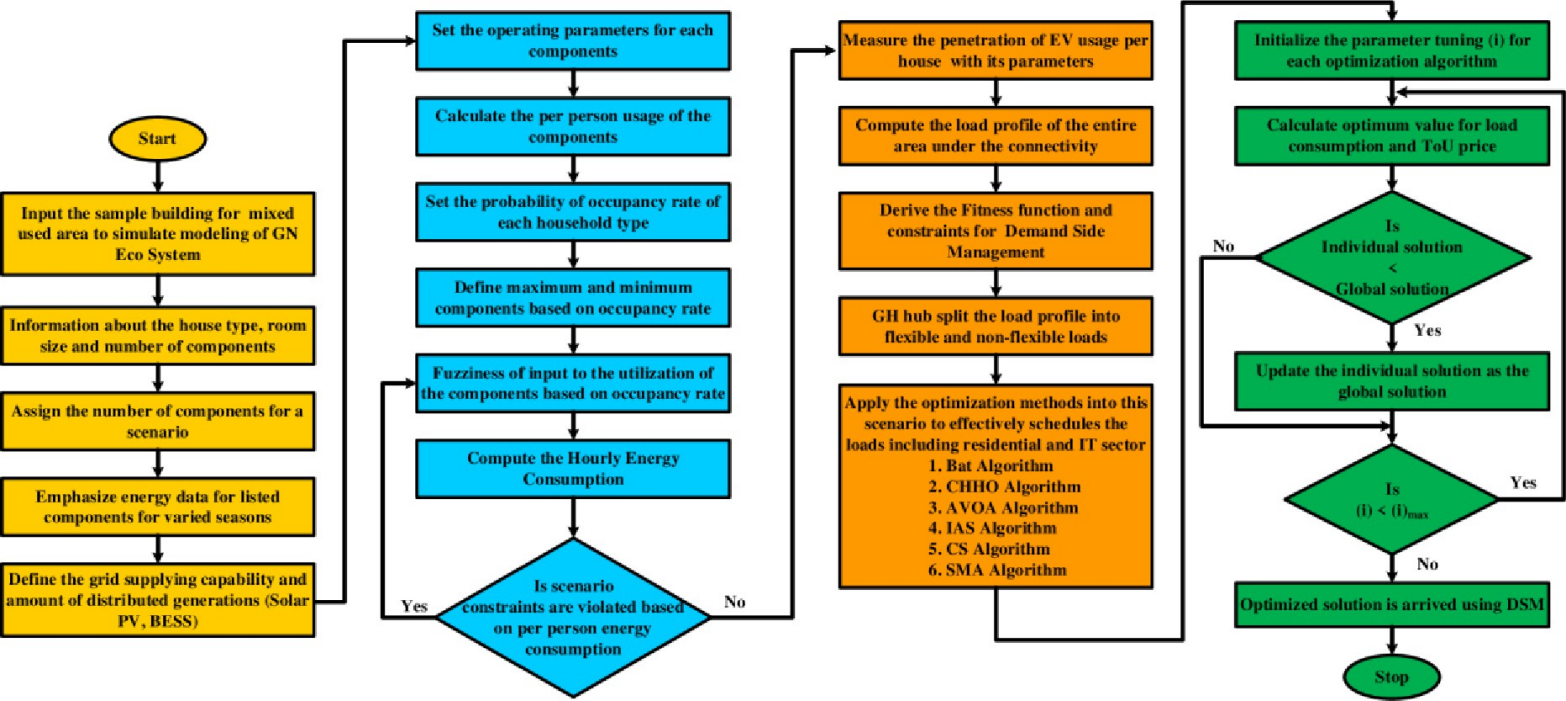
DSM optimization method implementation flowchart.

There are different optimization methods that have been used for developing the DSM with the modified constraints. The detailed different optimization algorithms implementation for DSM model is shown in the flowchart Fig 7, along with the steps involved in the execution and the corresponding pseudocode were discussed detailed as follows:

#### A. Bat optimization algorithm

The BOA was developed by Xin-She Yang in 2010 [25]. It simulates the foraging behavior of bats, which use echolocation to detect prey, avoid obstacles, and locate their roosting crevices in the dark. Bats emit a sound wave and listen to the echo that bounces back from surrounding objects. The BA uses the concept of frequency, velocity, and loudness to simulate bats’ search for optimal solutions in a problem space.

The behaviors of algorithms are:

- Frequency (f_i_) Bats use varying frequencies to search and home in on prey. In BA, this corresponds to the rate of change in solutions.
- Velocity (v_i_): Bats fly with varying speeds to cover different ranges. This correlates with the speed of convergence in the search space.
- Loudness (A_i_): This aspect is utilized to adjust the exploration and exploitation balance during the search.
- Pulse Emission Rate (r_i_): Determines the rate of pulse emission for echolocation, analogous to the rate of local search around the current best solution.

The various steps involved in the establishment of the algorithm are:

Step 1: Initialization: Generate the initial population of bats, each with a random position (X_i_) and velocity (v_i_) within the defined constraints.
Step 2: Fitness Evaluation: Calculate the fitness of each bat, which is the peak demand D_peak_ for its corresponding DSM schedule.
Step 3: Loop Until Convergence: For each iteration, update the bats’ positions and velocities based on the following rules:
  - Frequency Update:

$$f_{i}=f_{\min}+(f_{\max}f_{\min})\beta$$ (21)

Where *β* is a random vector drawn from a uniform distribution.

- Velocity Update:

$$(v_{i}^{\left(t+1\right)}=v_{i}^{\left(t\right)}+(X_{i}X_{*})f_{i}$$ (22)

Where X_*_ is the current global best location which has the lowest peak demand found so far.

- Position Update:

$$X_{i}^{\left(t+1\right)}=X_{i}^{\left(t\right)}+v_{i}^{\left(t+1\right)}$$ (23)

- Local Search: If a random number is greater than r_i_ select a solution among the best solutions and perform a random walk.
- Loudness and Pulse Emission Rate Update: Decrease A_i_ and increase r_i_ as the iterations proceed.
- Selection: If the new solutions have better fitness and a random number is less than the loudness, accept the new solutions and update X_*_ if applicable.
  Step 4: Loop until a predetermined condition is met with min D_peak_.

Pseudocode:

Algorithm: Bat Algorithm for Demand Side Management

Input: Population size N, Frequency bounds f_min_, f_max_, Loudness A_0_, Pulse rate r_0_, Max iterations Iter_max_

Output: Best Performing Bat position to optimal scheduling of appliances

1: Initialize the bat population X_i_ and velocities v_i_ for i = 1 to N

2: Define Pulse frequency f_i_ at x_i_

3: Initialize pulse rates r_i_ and the loudness A_i_

4: While (t < Iter_max_) or (convergence criteria is not met) do

5: for each bat i = 1 to N do

6:  Generate new solutions by adjusting frequency, and updating velocities and positions

7:  if (rand > r_i_) then

8:   Select a solution among the best solutions

9:   Generate a local solution around the selected best solution

10:  end if

11:  Generate a new solution by flying randomly

12:  if (rand < A_i_ && f(X_i_) < f(X_*_)) then

13:   Accept the new solution

14:   Increase r_i_ and reduce A_i_

15:  end if

16:  Rank the bats and find the current best X_*_

17: end for

18: t = t + 1

19: end while

**Fig 8:**
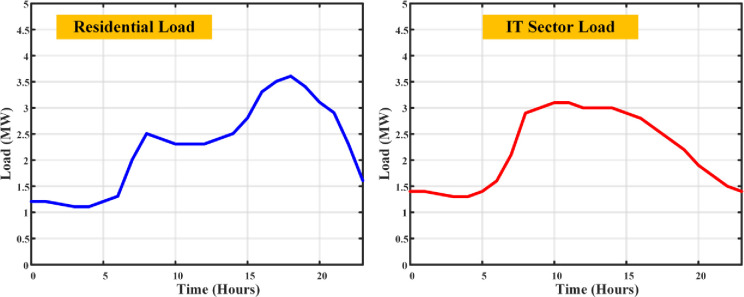
Load curves of residential and IT sector loads with BOA based DSM.

The execution of the BOA analyses each data of the appliances, loads and its state of operations, flexible loads, non-flexible loads and so on. The DSM has been achieved by the above steps and the results has plotted. The efficiency of the DSM using BO algorithm has been visualized from the Fig 8, where the load distribution is smooth as compared to the DSM.

#### B. Chaotic Harris Hawk Optimization (CHHO)

CHHO enhances the standard HHO by integrating chaotic maps, which are deterministic, yet unpredictable systems that help in diversifying the search pattern [26]. This aids in preventing premature convergence and ensuring the global optimum is reached. The metaphor of the Harris’ hawk’s cooperative hunting behavior is translated into mathematical models that mimic chasing, encircling, and attacking the prey, analogous to searching for the optimal solution in the problem space.

The algorithms behaviors are:

- Exploration Phase: Hawks randomly search for prey based on several chaotic exploration strategies, improving the diversity of the solutions.
- Exploitation Phase: Hawks perform several attacking strategies, including surprise pounce (hard besiege), rapid dives (soft besiege), and team chasing based on the prey’s escape patterns.
- Chaos Theory: Introduction of chaotic variables helps in maintaining the diversity of the population and avoiding local optima.

The various steps involved in the establishment of the algorithm are:

Step 1: Initialization: Initialize the population and the corresponding fitness of each hawk based on the DSM problem’s constraints.
Step 2: Loop Until Convergence: For each iteration, perform the following steps:
  - Chaos Update: Integrate a chaotic sequence into the search process to enhance exploration and exploitation.
  - Exploration and Exploitation: Hawks switch between exploration and exploitation phases, where the behavior is modeled by mathematical equations incorporating the chaotic variables.
  - Update Hawks’ Positions: Depending on the phase, update the hawks’ positions either randomly (exploration) or based on the best solution found so far (exploitation).
  - Local and Global Searches: Perform local and global searches based on the position of the prey, which represents the current best DSM strategy.
  - Fitness Evaluation: Evaluate the fitness of each hawk’s position and update the best solution if a better one is found.
Step 3: Is Last iteration, if yes then print the best solution, else then return to step 2.

Pseudocode:

Algorithm: Chaotic Harris Hawk Optimization for Demand Side Management

Input: Population size N, Max iterations Iter_max, Chaotic map settings

Output: Predominant Harris Hawk Position for reduced peak load values

1: Initialize the hawk population X_i and their fitness for i = 1 to N

2: Initialize chaotic variables and select a chaotic map

3: While (t < Iter_max) or (convergence criteria is not met) do

4: if (Exploration phase) then

5:  Update the positions of hawks using chaotic variables

6: else if (Exploitation phase) then

7:  Update positions based on the best solution and chaotic variables

8: end if

9: Evaluate the fitness of each hawk

10: Update the best solution if a better solution is found

11: Apply chaos to the system to ensure diversity

12: t = t + 1

13: end while

**Fig 9:**
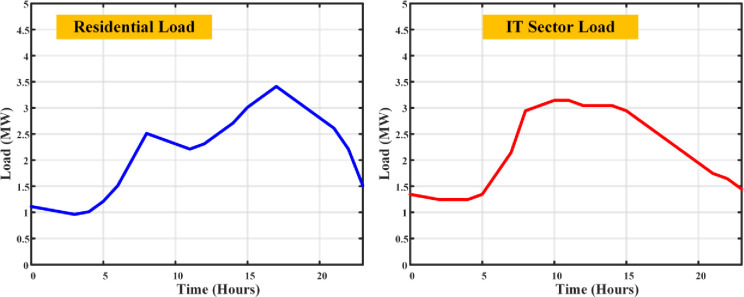
Load curves of residential and IT sector loads with CHHO based DSM.

The load profile curve achieved from the DSM using CHHO algorithm is shown in Fig 9. This algorithm operates the DSM with the peak load of 3.4MW at residential and 3.1MW at the IT sector Loads respectively, whereas the normal DSM with the defined EV constraints operated with 4.3MW at residential side and 3.5MW at the IT sector side.

#### C. African vulture optimization algorithm

AVOA was inspired by the intelligent foraging strategy of African vultures, known for their efficient scavenging and exceptional flight dynamics [27]. These birds utilize various flight patterns to maximize the probability of finding food with minimum energy consumption. The AVOA translates these behaviors into optimization processes to efficiently search for global optima within a given search space.

The algorithms behaviors are:

- Search for Carcass: Mimicking vultures spotting a carcass from high altitudes.
- Explorative Flight: Representing the wide-ranging search for new feeding opportunities.
- Exploitative Flight: Symbolizing the intense local search when a food source is known.
- Energy Consumption: Analogue to the computational effort required for the optimization process.
- Flight Adjustments: The adaptation of search patterns based on success rates and environmental cues.

The various steps involved in the establishment of the algorithm are:

Step 1: Initialization: Define initial positions for the vultures, representing possible DSM strategies within the constraints of the problem.
Step 2: Fitness Evaluation: Evaluate each vulture’s position for fitness, which is the peak demand D_peak_ for the DSM strategy it represents.
Step 3: Optimization Loop: For each iteration, execute the following:
  - Determine Search Phase: Depending on the success of the previous iterations, decide whether to perform explorative or exploitative flights.
  - Update Positions: Adjust the vultures’ positions based on the chosen search phase, factoring in energy levels and the perceived distance to the carcass (optimal solution).
  - Local Search (if necessary): Conduct a local search around the current best positions to refine solutions.
  - Energy Consumption: Simulate the energy consumption of vultures, adapting their flight dynamics over time.
  - Selection: Keep the best solutions and discard those with higher peak demands.
Output the Optimal Strategy: If the maximum number of iterations is reached or if convergence is achieved, end the optimization process.

Pseudocode:

Algorithm: African Vulture Optimization Algorithm for DSM

Input: Population size N, Max iterations Iter_max

Output: Top-Scoring Vulture Position for optimal solution vector

1: Initialize the positions of the vultures

2: Evaluate the initial fitness of the vultures

3: While (not converged and iterations < Iter_max) do

4: for each vulture i do

5:  Decide the search phase (explorative or exploitative)

6:  Update the position of vulture i based on the search phase

7:  Perform local search if required

8:  Evaluate the new position’s fitness

9:  if (new fitness better than current fitness) then

10:   Update the position to the new position

11:  end if

12: end for

13: Update the global best position if necessary

14: iterations++

15: end while

**Fig 10:**
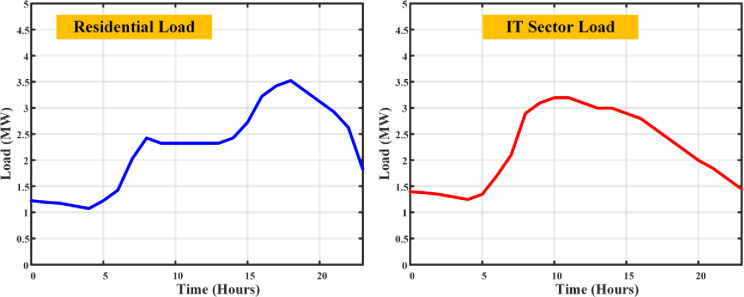
Load curves of residential and IT sector loads with AVOA based DSM.

The load profile curve achieved from the DSM using AVOA algorithm is shown in Fig 10. This algorithm operates the DSM with the peak load of 3.5MW at residential and 3.2MW at the IT sector Loads respectively.

#### D. Chaotic interactive autodidact school algorithm

The CIAS Algorithm is inspired by the self-learning and interactive teaching processes found in academic environments [28]. It emulates the way students (solutions) learn both autonomously and through interaction with their peers (other solutions). By incorporating chaotic maps, the algorithm gains an ability to avoid local minima and ensures global optimization through enhanced exploration.

The algorithms behaviors are:

- Knowledge Sharing: Analogous to global optimization, where information is shared among the population to reach the best solution.
- Self-Study: Represents local search or refinement of the current solution.
- Teacher-Student Interaction: Informs the balance between exploration (global search) and exploitation (local search).
- Chaotic Sequences: Introduce unpredictability, which enables the algorithm to escape local optima and improve the diversity of solutions.

The various steps involved in the establishment of the algorithm are:

Step 1: Initialization: Generate the initial population and assign initial knowledge levels to each student (solution).
Step 2: Fitness Evaluation: Calculate the fitness (electrical load profile) for each student’s DSM strategy.
Step 3: Loop Until Convergence: Iteratively perform the following steps, utilizing chaotic sequences to guide the search:
  - Knowledge Update: Update each student’s knowledge based on their own study and interactions with other students.
  - Chaotic Local Search: Apply chaotic maps to perform a local search around the current solution to avoid premature convergence.
  - Teacher-Student Interaction: Select the best solution (teacher) to guide the other students, ensuring knowledge diffusion.
  - Self-Study Process: Students independently refine their solutions based on personal learning rates.
  - Global Knowledge Sharing: Share the best solution’s information with the entire population, emulating a global update.
  - Selection: Select the best students (solutions) based on the reduced peak demand and ensure diversity through chaotic behavior.
Output the Best Solution: Continue the iteration process until the predefined condition involving the minimum of D_peak_ is achieved.

Pseudocode:

Algorithm: Chaotic-based IAS Algorithm for DSM

Input: Population size N, Max iterations Iter_max_, Chaotic map parameters

Output: Foremost Learner Solution for DSM matrix

1: Initialize the student population and their knowledge levels

2: Define a chaotic map and initialize its parameters

3: While (t < Iter_max_) or (convergence criteria is not met) do

4: Evaluate the fitness of each student

5:  for each student i = 1 to N do

6:   Update student’s knowledge using chaotic local search

7:   Perform teacher-student interaction to share best solution

8:   Carry out self-study to refine student’s knowledge

9:   Apply global knowledge sharing

10:   if the student’s new strategy results in a lower peak demand, then

11:    Update the student’s knowledge to this new strategy

12:   end if

13:  end for

14: t = t + 1

15: Update chaotic map parameters

16: end while

**Fig 11:**
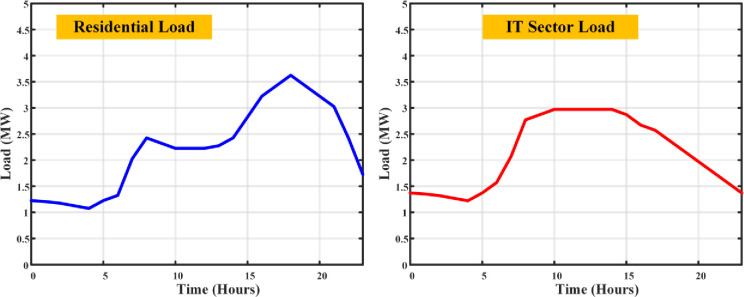
Load curves of residential and IT sector loads with CIAS based DSM.

The load profile curve achieved from the DSM using IASA algorithm is shown in Fig 11. This algorithm operates the DSM with the peak load of 3.6MW at residential and 3MW at the IT sector Loads respectively.

#### E. Cuckoo search algorithm

Developed by Xin-She Yang and Suash Deb in 2009, the CS algorithm is inspired by the obligate brood parasitism of some cuckoo species [29]. These cuckoos lay their eggs in the nests of other host birds. If the host bird detects the alien eggs, it will either throw them away or abandon its nest. The CS algorithm mimics this process using a balanced combination of local and global random walks, controlled by a switching parameter.

The algorithms behaviors are:

- Lévy Flights: This is a type of random walk, which the CS algorithm uses for global exploration, enabling efficient searching over a large space.
- Discovery Rate (P_a_): The probability of a host bird discovering a cuckoo egg, analogous to the rate of local exploitation in the search space.

Fixed Egg Laying Radius: Represents the area or neighborhood around a solution where new solutions (eggs) are laid.

The various steps involved in the establishment of the algorithm are:

Step 1: Initialization: Generate the initial nests, each representing a DSM schedule.
Step 2: Fitness Evaluation: Calculate the fitness of each nest based on the peak load(D_{peak}) for its DSM schedule.
Step 3: Loop Until Convergence: For each generation, perform the following steps:
  - Get a Cuckoo Randomly: Generate a new solution (X_i_) using Lévy flights for a randomly chosen cuckoo (nest).
  - Choose a Nest Randomly: Choose a nest randomly and compare its fitness with (X_i_).
  - Apply Discovery Rate: With a probability (Pa), determine if the eggs are discovered. If discovered, the nest is abandoned, and a new one is built (replace the solution in the nest with (X_i_) if (X_i_) is better).
  - Keep Best Nests: Retain the best nests and carry out local searches around them.
  - Abandon Worse Nests: A fraction (P_a_) of the worse nests are abandoned, and new ones are built at new locations via Lévy flights.
  - Rank the Nests and Find the Best: Rank the solutions/nests and find the current best nest.
  - Termination Check: If the maximum generations are reached or convergence criteria are met, terminate the algorithm.
Output the Best Solution: Continue executing the previous step until the specified termination criteria are satisfied.

Pseudocode:

Algorithm: Cuckoo Search for Demand Side Management

Input: Number of nests N, Discovery rate P_a_, Max generations Gen_max_

Output: Principal Cuckoo Nest Position for Peak Load Reduction

1: Initialize nests Xi randomly for i = 1 to N

2: Evaluate the fitness of each nest

3: while (gen < Gen_max_) do

4: Get a cuckoo (i.e., solution) randomly by Lévy flights

5: Choose a nest j randomly and evaluate its fitness

6: if (fitness (Xi) > fitness (Xj)) then

7:  Replace j with the new solution Xi

8: end if

9: Abandon a fraction P_a_ of worse nests and build new ones

10: Keep the best solutions (nests) and perform local searches

11: Rank the nests and find the current best

12: gen = gen + 1

13: end while

14: Output the best nest which is the DSM schedule

**Fig 12:**
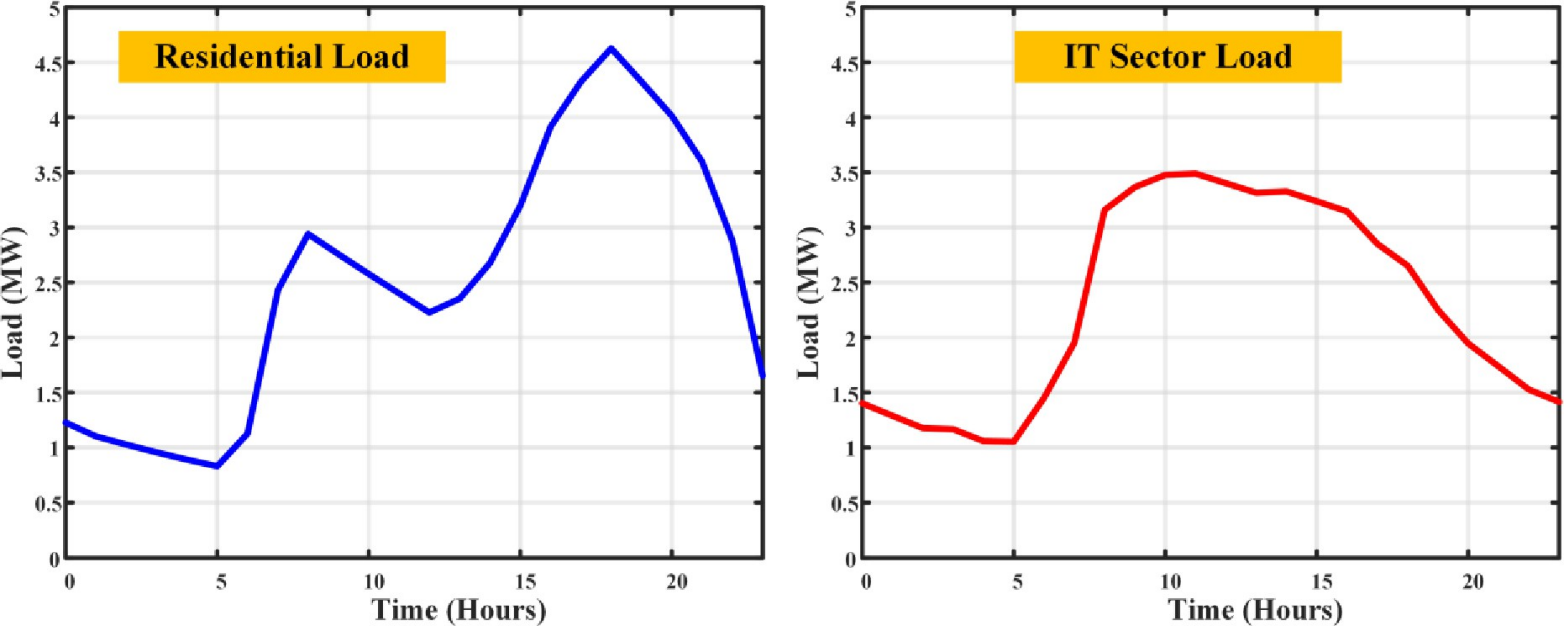
Load curves of residential and IT sector loads with CS algorithm based DSM.

The load profile curve achieved from the DSM using CS algorithm is shown in Fig 12. This algorithm operates the DSM with the peak load of 4.6MW at residential and 3MW at the IT sector Loads respectively.

#### F. Slime mould algorithm

The SMA, inspired by the slime mould’s ability to find the shortest path to food sources, is an effective method for solving complex optimization problems [30]. The algorithm mimics the oscillation pattern of slime mould in nature, which dynamically adjusts its body to optimize nutrient intake while minimizing exposure to threats.

The algorithms behaviors are:

- Oscillation Pattern: Slime mould changes its shape rhythmically, which is modeled in SMA to search through the solution space.
- Positive Feedback: As in nature, when a particular path is beneficial, the mould strengthens that path, akin to intensifying the search in a promising region.
- Balanced Exploration and Exploitation: The algorithm maintains a balance between diversifying the search (exploration) and intensifying the search around the promising solutions (exploitation).

The various steps involved in the establishment of the algorithm are:

Step 1: Initialization: Generate an initial population of slime moulds with random positions within the permissible DSM strategy space.
Step 2: Fitness Evaluation: Evaluate the fitness of each slime mould based on the objective function (D_peak_).
Step 3: Loop Until Convergence: For each iteration, perform the following steps:
  - Update Positions: Each slime mould updates its position according to the fitness of its current position and the fitness of other moulds, moving towards better positions.
  - Oscillation Phase: Simulate the rhythmic contraction and expansion of slime mould to explore the search space.
  - Positive Feedback Mechanism: If certain DSM strategies yield lower peak loads, reinforce those strategies among the population.
  - Adaptation: Adjust the search strategy based on the stochastic oscillation of the agents.
  - Selection: Retain the best-found solutions and discard the less effective ones.
Output the Best Solution: Check if it’s the final iteration. If it is, display the best-found solution; if not, proceed back to the third step.

Pseudocode:

Algorithm: Slime Mould Algorithm for Demand Side Management

Input: Population size N, Maximum iterations T_max_, Search Space Dimensions

Output: Slime Mould Pathway for load management pattern

1: Initialize the slime mould population X_i_ for i = 1 to N

2: Evaluate the fitness of each slime mould

3: Determine the best solution X_best_

4: While (t < T_max_) do

5: for each slime mould i do

6:  Update the position of slime mould i towards the best solution

7:  Oscillate to explore the search space

8:  Evaluate the updated fitness

9:  If the new position is better, update the individual and global best

10: end for

11: t = t + 1

12: end while

13: Return the optimized DSM schedule X_best_

**Fig 13:**
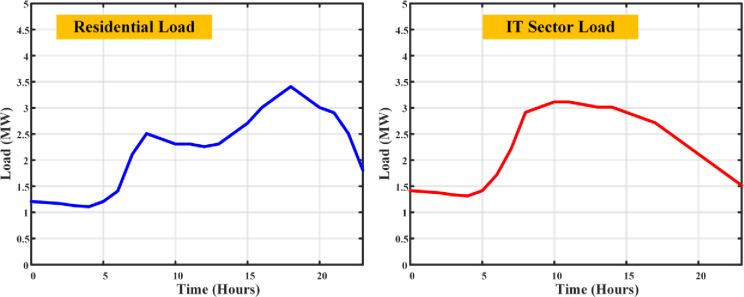
Load curves of residential and IT sector loads with SMA based DSM.

The load profile curve achieved from the DSM using SMA algorithm is shown in Fig 13. This algorithm operates the DSM with the peak load of 3.4MW at residential and 3.1MW at the IT sector Loads respectively.

## 5. Comparative analysis

In residential and IT sectors, energy demand patterns can vary significantly. DSM introduces a framework for intelligently adjusting these patterns, ensuring that energy consumption aligns more closely with sustainable practices. DSM strategies and algorithms have achieved significant peak load reduction, leading to a more balanced energy consumption pattern. As shown in the Table 1, baseline energy demand without DSM gives a peak load of 4.67 MW and a low of 0.47 MW. In the residential sector, there is a significant 4.2 MW variance between peak and low demand. For the IT sector, using general DSM formulas, the peak demand is 4.23 MW and the low demand is 0.99 MW, resulting in a 3.24 MW difference. Generic DSM model reduce variation between residential and IT sectors. The peak load is reduced to 4.52 MW for residential and 3.61 MW for IT. This decreases the peak to low difference to 3.39 MW for residential and 2.44 MW for IT, improving alignment of energy consumption with availability. Various advanced algorithms were evaluated for their performance, with the BAT Algorithm reducing the peak-to-low difference to 2.44 MW in the residential sector and 1.64 MW in the IT sector. The AVOA and SMA algorithms also show improvements, with the SMA algorithm achieving a residential peak-to-low difference of 2.36 MW. However, the most effective algorithm remains the CHHO algorithm, with peak-to-low differences of 2.36 MW for residential and 1.62 MW for IT. The CS algorithm achieves a peak-to-low difference of 3.6 MW in the residential sector and 2.56 MW in the IT sector, which is not as effective as CHHO but significantly better than the baseline DSM.

**Table 1:** Summarized load characteristics of each algorithm.

S.NO	Different Algorithms	Residential Load (MW)			IT Sector Load (MW)		
		Res_Peak_	Res_Low_	Peak to Low	IT_Peak_	IT_Low_	Peak to Low
1	Without DSM	4.67	0.47	4.2	4.23	0.99	3.24
2	DSM	4.52	1.13	3.39	3.61	1.17	2.44
3	BOA	3.76	1.32	2.44	3.33	1.69	1.64
4	CHHO	3.69	1.11	2.58	3.45	1.41	2.04
5	AVOA	3.73	1.37	2.36	3.21	1.59	1.62
6	CIAS	3.82	1.37	2.45	3.29	1.38	1.91
7	CS	4.6	1	3.6	3.76	1.2	2.56
8	SMA	3.66	1.4	2.46	3.32	1.54	1.78

**Fig 14:**
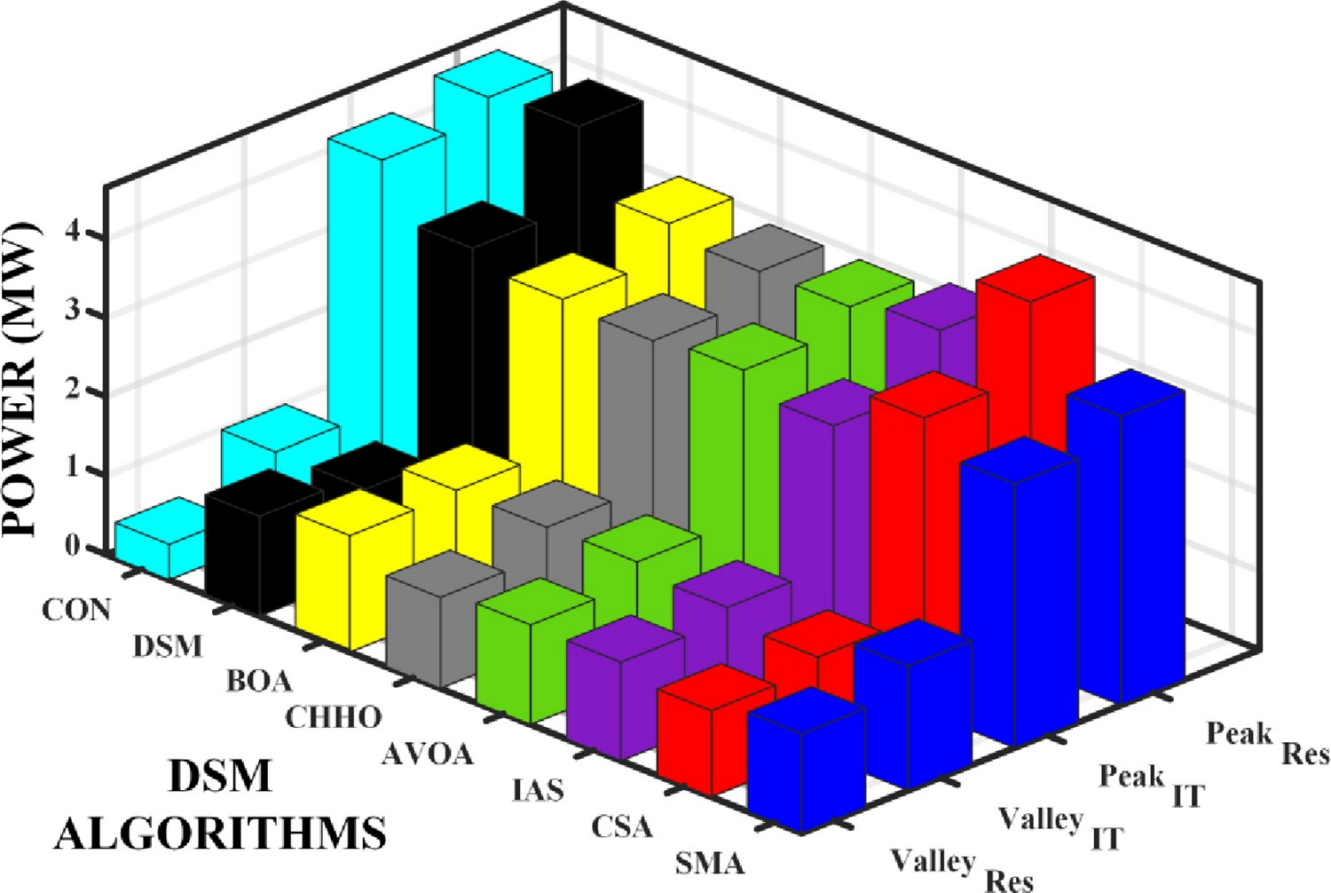
Performance comparison chart of the different DSM algorithms.

**Fig 15:**
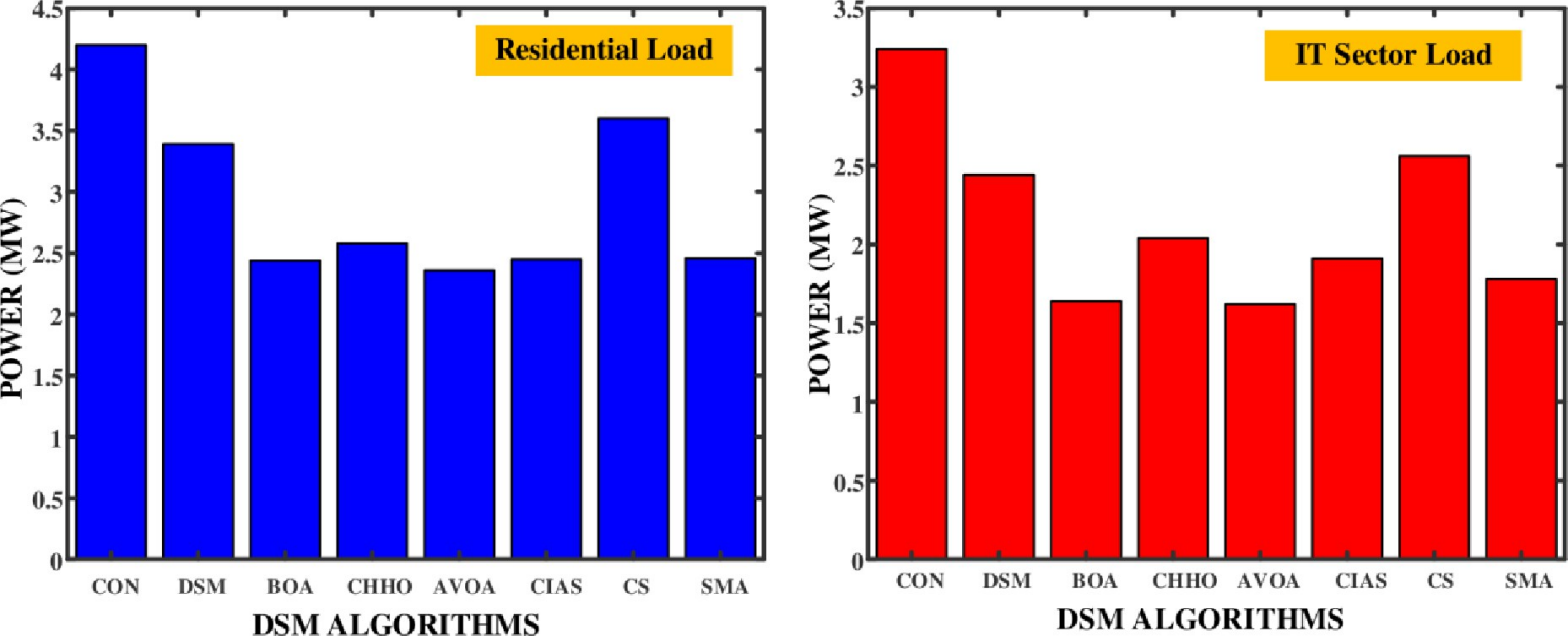
Efficiency improvement in terms of different between peak to valley.

The reduction in peak loads and the corresponding increase in low loads with DSM implementation hint at a significant improvement in both sectors’ energy consumption profiles. The comparative chart of the peak load, least load in residential and IT sector loads are shown in Fig 14. The efficiency of the algorithm can be evaluated by measuring the difference between the peak demand and least demand as shown in Fig 15.

**Fig 16:**
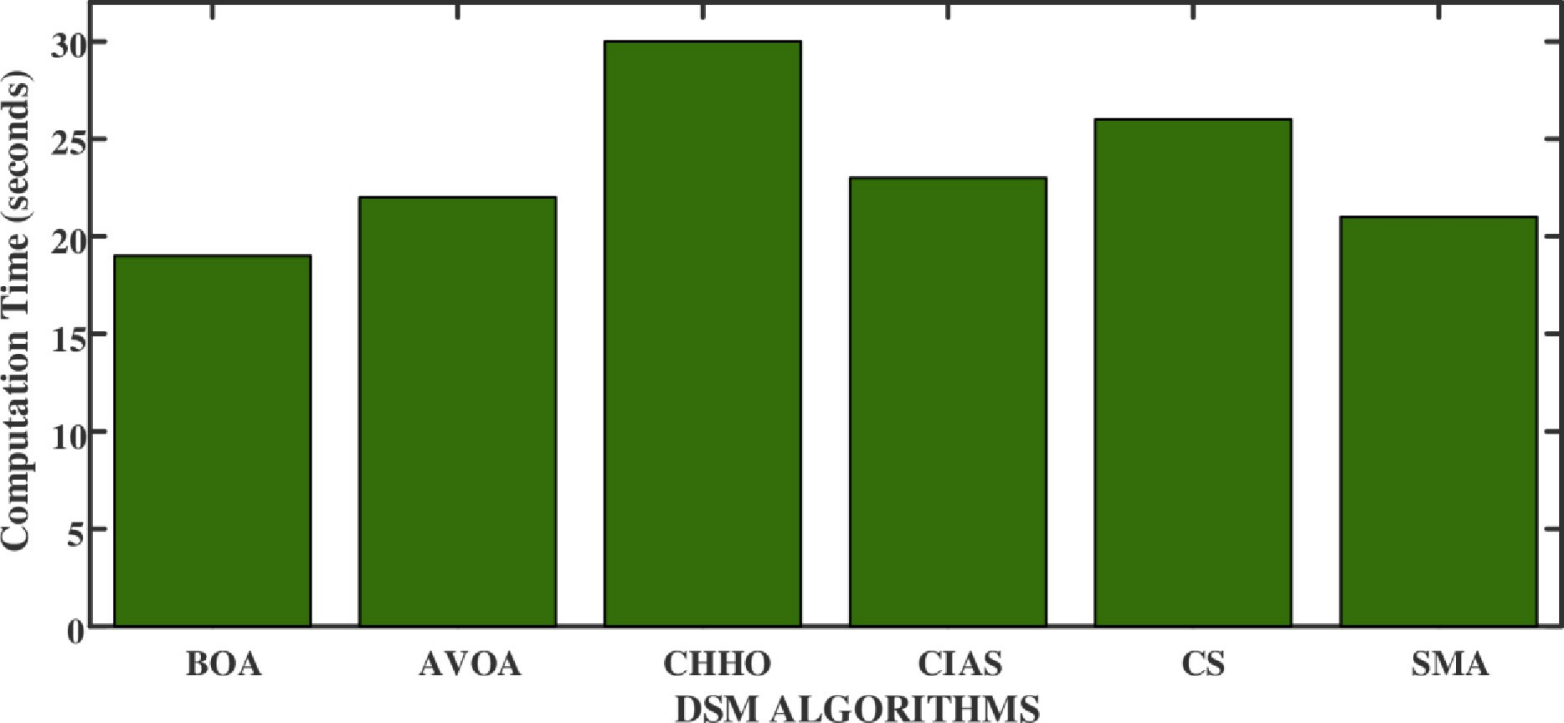
Comparison chart of computation time.

The computation times of algorithms (BOA, AVOA, CS, CHHO, CIAS, and SMA) were measured and compared as shown in Fig 16. The algorithms were run on a high-performance Intel i7 13^th^ gen 1335 processor with 16 GB ram computing setup under identical conditions to determine the best method in terms of result and speed.

**Fig 17:**
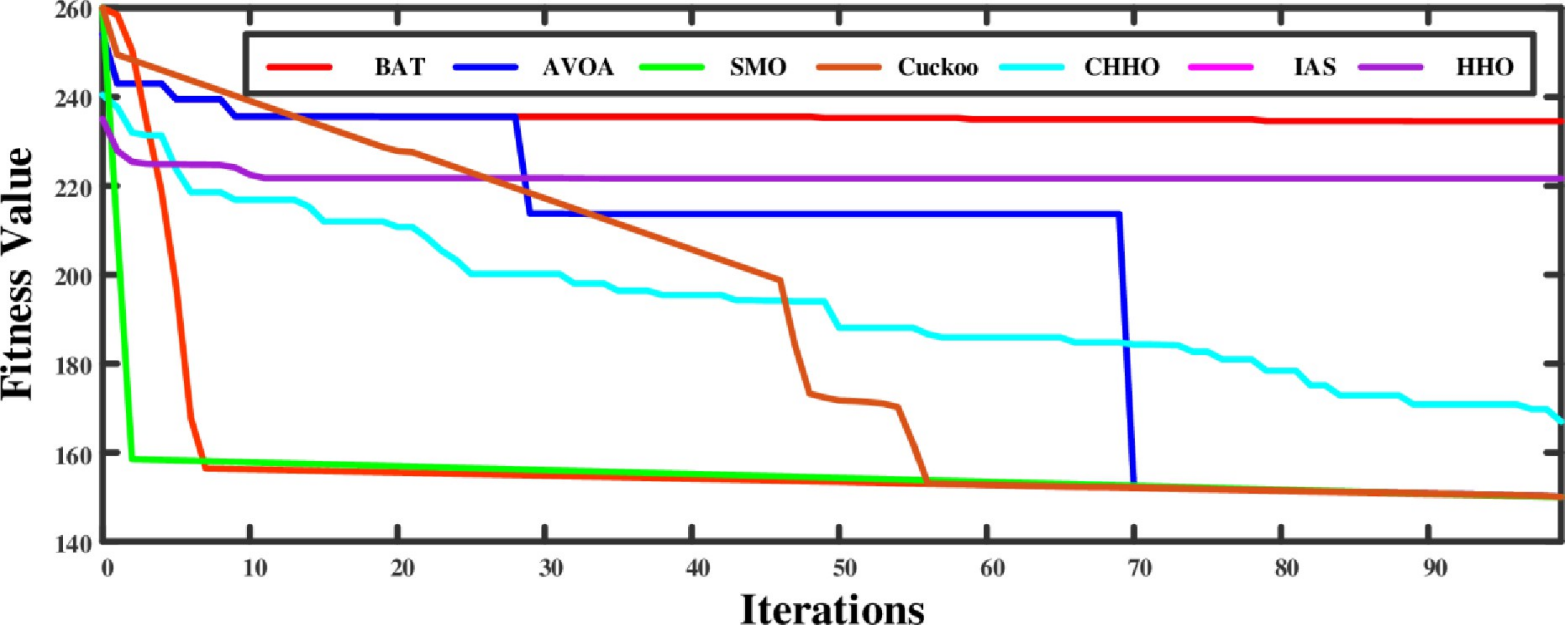
Convergence curve of each DSM algorithms.

Fig 17 shows the convergence curves of each algorithm have been plotted as fitness values vs iteration. Using this plot, it can be inferred that CHHO is the most efficient in terms of least convergence happened at 9^th^ iteration and maintaining a low fitness value of 155, which might suggest finding the global minimum. AVOA also shows quick convergence, but it plateaus early, which may suggest a risk of not finding the global minimum if it isn’t reached early in the search. Unlike the other algorithms, the CS and BOA exhibit a gradual convergence. This characteristic proves beneficial when dealing with a rugged search space of GN and the potential for being trapped in local minima. On the other hand, the SMO consistently maintains a low value, indicating that it initially performs well but does not show significant improvement. This could be attributed to either finding a near-optimal solution early or being unable to escape a local minimum if the solution is not optimal.

## 6. Conclusion

The work has demonstrated that the integration of EVs into the power grid, while beneficial for reducing carbon emissions, poses substantial challenges for load management. The proposed optimization-based DSM algorithm effectively addresses these challenges, exhibiting significant improvements in managing the daily demand and load curve of the system. Comparative analysis with other algorithms has shown that this proposed approach not only reduces peak loads but also facilitates a more stable and balanced grid by effectively shifting EV charging loads. The implications of these findings are twofold: firstly, it highlights the potential of advanced DSM strategies to enhance grid management in the era of EV expansion; secondly, it provides a framework for utilities and energy managers to incorporate optimization-based DSM algorithms into their operational strategies, including EV charge scheduling. The CHHO Algorithm’s impressive performance in the IT realm, contrasted with the Slime Mould Algorithm’s remarkable efficiency in residential areas, underscores their respective proficiency. The distinct effectiveness of each algorithm across varied sectors underscores the imperative for customized solutions in orchestrating load management tactics. Delving into the dynamics of DSM algorithms unveils profound insights into their ability in forming energy consumption curves, with the CHHO performing better than other algorithms in residential loads whereas SMA performs better alongside CHHO in the IT sector load. However, CHHO shows a better convergence rate compared to SMA and other algorithms, performing well for objective functions and constraints when considering all loads in the GN system. The study suggests future investigations into the scalability of DSM approaches across diverse geographical and demographic contexts, potentially leading to widely applicable and reliable energy management solutions for various electric grid scenarios, including versatile EV charge scheduling methods.
